# Species Distribution and Antifungal Susceptibility Patterns of Invasive Candidiasis in a Belgian Tertiary Center: A 7-Year Retrospective Analysis

**DOI:** 10.3390/jof11060465

**Published:** 2025-06-19

**Authors:** Sarah Cugnata, Rosalie Sacheli, Nathalie Layios, Marie-Pierre Hayette

**Affiliations:** 1 Department of Clinical Microbiology, University Hospital of Liege, 4000 Liege, Belgium; sarah.cugnata@gmail.com; 2 Department of Clinical Microbiology, Center for Interdisciplinary Research on Medicines (CIRM), University Hospital of Liege, 4000 Liege, Belgium; r.sacheli@chuliege.be; 3 National Reference Center for Mycosis, University Hospital of Liege, 4000 Liege, Belgium; 4 Intensive Care Department, University Hospital of Liege, 4000 Liege, Belgium; nathalie.layios@chuliege.be

**Keywords:** invasive candidiasis, epidemiology, resistance

## Abstract

Candidiasis is a major fungal infection worldwide, with invasive forms linked to high morbidity and mortality. The emergence of azole resistance in *Candida parapsilosis* causing candidemia led us to examine the epidemiology and antifungal susceptibility of *Candida* species at the University Hospital of Liège between January 2017 and December 2023. A total of 916 isolates from blood or sterile body fluids, tissues, and abscesses were analyzed. Species identification was performed using MALDI-TOF MS and antifungal susceptibility testing via Sensititre YO10 AST was interpreted according to the CLSI guidelines. *Candida albicans* remained the predominant species (56%), followed by *Nakaseomyces glabratus* (19%), *Candida parapsilosis* (8%), and *Candida tropicalis* (7%). No significant shift toward non-*albicans Candida* species (NAC) was observed even during the COVID-19 pandemic, supporting the use of narrow-spectrum empirical therapy in selected patients. Fluconazole susceptibility was high in *C. albicans* (98.8%), whereas *N. glabratus* and *C. tropicalis* showed high resistance rates with 10.1% and 16.9%, respectively. *C. parapsilosis* showed stable fluconazole susceptibility across the study period. Echinocandins demonstrated excellent activity (95.6–100%), and amphotericin B was effective against nearly all isolates. This seven-year surveillance at the University Hospital of Liège confirms that while *C. albicans* remains the predominant and highly susceptible species, rising azole resistance in non-*albicans Candida*—particularly *N. glabratus* and *C. tropicalis*—highlights the critical need for ongoing local epidemiological monitoring to guide effective and targeted antifungal therapy.

## 1. Introduction

Over the past decades, the incidence of invasive fungal infections has increased dramatically in immunocompromised patients, including non-neutropenic critically ill adults [1,2]. A number of factors are associated with this rise in incidence, including therapeutic advances in hematology and intensive care such as immunosuppressive treatments, chemotherapies, invasive techniques (e.g., intravascular catheters, drainage devices, etc.), and the use of broad-spectrum antibiotic therapy [3,4]. Among these invasive fungal infections, candidiasis is one of the main fungal infections worldwide in hospitalized patients behind invasive aspergillosis and chronic pulmonary aspergillosis [1]. The term invasive candidiasis (IC) is used to describe candidemia (bloodstream infection) but also deep-seated organ infections which result from the dissemination of *Candida* species to normally sterile body sites, such as the abdomen, peritoneum, eye, or bone [5].These two clinical manifestations may occur independently or concomitantly [6]. In terms of incidence, there are over 600,000 cases of candidemia per year with an estimated overall mortality of 30–40% and over 900,000 cases of invasive candidiasis without candidemia per year worldwide [1].

More than 15 species of *Candida* can cause invasive infections. *C. albicans*, *Nakaseomyces glabratus* (formerly *C. glabrata*), *C. parapsilosis*, *C. tropicalis*, and *Pichia kudriavzevii* (formerly *C. krusei*) are the most frequently implicated. In recent years, although *C. albicans* remains the leading cause of both candidemia and other forms of invasive candidiasis, the epidemiology of invasive candidiasis has notably shifted with a growing prevalence of non-*albicans Candida* (NAC) species that exhibit decreased susceptibility to commonly used antifungal agents. This shift is multifactorial and appears to be driven by variations in antifungal usage, patient populations, and hospital practices.

Importantly, significant epidemiological differences are observed across Europe. In Northern European countries (e.g., Denmark, the UK, Sweden, and the Netherlands), *C. albicans* remains the most prevalent, followed by *N. glabratus*, which accounts for up to 30% of cases in some cohorts [7]. In contrast, Southern European and Mediterranean countries (e.g., Italy, Spain, Greece) more frequently report *C. parapsilosis* as the second most common species, particularly in ICUs and neonatal units [2,8,9,10,11].

Alongside these shifts in species distribution, resistance to fluconazole is emerging as a major challenge. In Northern Europe, fluconazole resistance among *N. glabratus* isolates reaches 10–11% [7,12], while in Southern Europe, increasing resistance in *C. parapsilosis* has been associated with clonal outbreaks in hospital settings. Since the COVID-19 pandemic, several European hospitals have reported the nosocomial transmission of fluconazole-resistant *C. parapsilosis* strains carrying *ERG11* gene mutations—most notably the Y132F substitution [13]. Moreover, novel resistance mechanisms such as *MRR1* mutations and efflux pump overexpression have been recently described, further complicating treatment options [14].

At the same time, the emergence and global spread of multidrug-resistant *C. auris* has raised further concern, with outbreaks reported in various healthcare settings worldwide. These developments highlight the need for continued epidemiological surveillance to monitor species distribution and antifungal resistance patterns, which are critical for guiding both empirical and targeted antifungal therapy and for preventing nosocomial transmission [2,15,16].

The growing resistance among *Candida* species, in addition to sporadic outbreaks of fluconazole-resistant *C. parapsilosis* in neighboring countries, especially during the COVID-19 pandemic [13,14], led us to conduct a retrospective overview of our local epidemiology at the University Hospital of Liege (CHU Liege) over the last 7 years on strains isolated from patients with candidemia or invasive candidiasis. The aim was to identify temporal changes and the current distribution of *Candida* species in parallel with their respective antifungal resistance rates. Ultimately, this retrospective study could serve local antifungal stewardship purposes either in the empiric or the targeted therapy setting.

## 2. Materials and Methods

This study was a retrospective laboratory data-based study designed to investigate the epidemiology of *Candida* species at CHU Liege between January 2017 and December 2023. Our institution is a 1038-bed academic hospital with tertiary medico-surgical activity including solid organ transplants (except lung transplants), the use of CAR-T cells and allogeneic marrow transplantation, one burn unit (6 beds), and 7 ICUs. For this purpose, selection was performed as follows: all yeast isolates retrieved from blood cultures, sterile body fluids, tissues, and abscesses for which antifungal susceptibility testing (AST) had been performed were included in this study. Yeast isolates from the gastrointestinal tract (biliary tract fluid) and catheters were also included. However, yeast isolates from sputum, bronchoalveolar lavage (BAL), urine, and the genital tract were considered colonizers and were therefore excluded from the analysis. The relevant infections were often associated with candidemia, which was included in the study from the outset.

### 2.1. Patients and Episodes

The clinical data collected included age, gender, ward location at the onset of candidiasis (with or without candidemia), underlying disease, comorbidities, the date of sample collection, the clinical site of isolation, and specimen type. Some patients experienced multiple episodes of candidiasis during the study period. Isolates of the same species with identical susceptibility profiles, obtained from the same patient within 30 days of the initial isolation, were considered part of the same episode of IC and were therefore excluded.

### 2.2. Isolation and Species-Specific Identification of Yeast Isolates

Blood samples were processed using the BACT/ALERT^®^ VIRTUO^®^ Microbial Detector System (bioMerieux, Marcy l’Etoile, France). Positive samples with yeasts observed on Gram stain examination were plated on Sabouraud Dextrose Agar with chloramphenicol (SGC2, bioMerieux Inc.) and incubated at 37 °C for 24–48 h. For other samples, Gram staining was performed upon receipt, followed by plating and incubation using the KIESTRA BD automated system (BD, Quebec City, QC, Canada). Colonies were identified using the Matrix-Assisted Laser Desorption/Ionization Time of Flight (MALDI TOF) Vitek^®^ MS (bioMérieux, Marcy l’Etoile, France). Obtained spectra were compared with the RUO BiotyperTM database (Bruker Daltonics, Ettlingen, Germany/Billerica, MA, USA) for yeast identification, considering identification with score values ≥ 2.

### 2.3. Antifungal Susceptibility Testing

Antifungal susceptibility testing (AST) was performed using Sensititre YO10 AST plates (Thermo Fisher Scientific, Waltham, MA, USA) according to the Clinical Laboratory Standards Institute (CLSI)-approved standard M27M44S for yeasts [17]. The antifungal drugs tested included amphotericin B (AMB), fluconazole (FLU), itraconazole (ITRA), voriconazole (VOR), caspofungin (CAS), and anidulafungin (ANI). Briefly 20 µL of a 0.5 McFarland inoculum suspension was added to YeastOne broth (Thermo Scientific, USA). Then, 100 µL of the suspension was distributed into each well of the AST plate. The plates were then incubated at 37 ± 2 °C for 24 h. Minimum inhibitory concentrations (MICs) were determined through visual examination using the Sensititre VIZION camera (Thermo Scientific, USA) and were interpreted using the CLSI M27M44S clinical breakpoints or M57S epidemiological cutoff values.

### 2.4. Statistical Analysis

Statistical analysis was performed using the R software (v. 4.4.2) and a Chi-square test was applied for comparisons between categorical variables and to assess differences in species distribution over the study period. Results were considered statistically significant when the *p*-value was <0.05.

## 3. Results

### 3.1. Temporal Trends and Distribution of Candida Species over Seven Years

**Figure 1:**
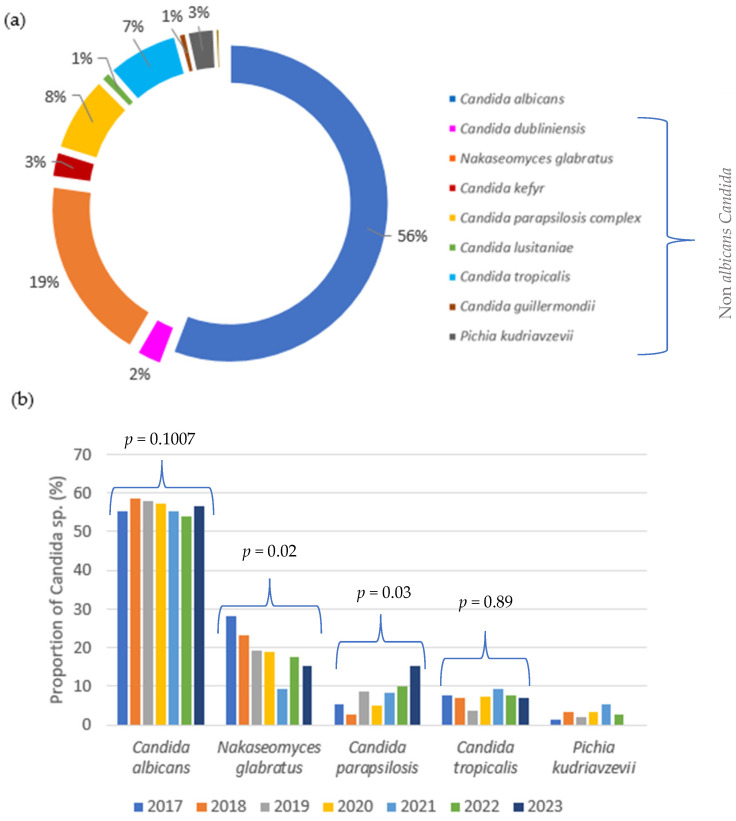
(**a**) Distribution of *Candida* species: proportion (%) of *Candida* species detected between 2017 and 2023. (**b**) Evolution of the *Candida albicans*/*NAC* ratio (%) per year over the 7-year study period.

A total of 916 yeast isolates were obtained from 795 patients and 13 *Candida* species were identified. *C. albicans* remained the most prevalent species (56%), followed by *N. glabratus* (19%), *C. parapsilosis sensu lato* (8%), *C. tropicalis* (7%), and *P. kudriavzevii* (3%). Other *Candida* species identified were *C. kefyr* (2.6%), *C. dubliniensis* (2%), *C. guilliermondii* (0.7%), *C. lusitaniae* (1%), *C. inconspicua* (0.4%), *C. norvegiensis* (0.1%), *C. pararugosa* (0.1%), and *C. pelliculosa* (0.1%), collectively accounting for 7% of NAC (Figure 1a). The *C. parapsilosis sensu lato* group included *C. parapsilosis sensu stricto* (70/71) and *C. metapsilosis* (1/71). Over the past 7 years (see Figure 1b and Supplementary Table S1), *C. albicans* has consistently accounted for more than 50% of isolates each year. Although a slight decreasing trend was observed, the ratio of *C. albicans* to non-*albicans Candida* (NAC) species remained stable. A significant decrease in the frequency of *N. glabratus* was observed, from 27.8% in 2017 to 15% in 2023 (*p* < 0.05). In contrast, the frequency of *C. parapsilosis* significantly increased from 5.3% to 15% over the same period (*p* < 0.05). This decrease in *N.glabratus* was compensated for by the increase in *C. parapsilosis*, contributing to the stable ratio of *C. albicans* to NAC species during the study period. The frequency of other species remained relatively stable, with only slight and statistically insignificant variations over the years (*p* > 0.05).

**Figure 2:**
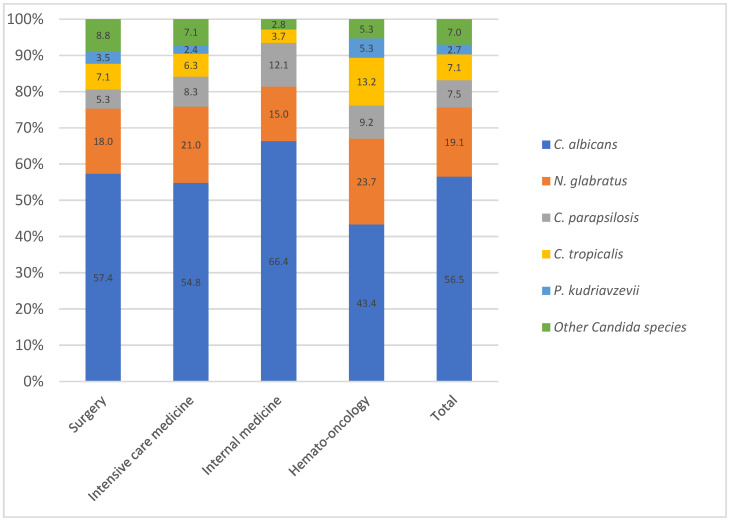
Distribution (%) of all *Candida* species according to ward.

The distribution of *Candida* species according to the patient ward is shown in Figure 2. The majority of isolates came from surgery (47%) with abdominal surgery representing the predominant origin. This was followed by isolates from ICUs (27%), including 55.5% from the surgical ICU, 12.6% from the general ICU, 12.6% from the medical ICU, 11.7% from the burns ICU, and 7.7% from the coronary ICU. The medical wards accounted for 12% of the samples, while the hemato-oncology wards accounted for another 8%.

The species distribution differed according to the patient population and risk factors, as shown in Figure 2. In hemato-oncology patients, *C. albicans* was isolated in 43.4% of the cases, *N. glabratus* in 23.7%, *C. parapsilosis* in 9.2%, and *C. tropicalis* in 13.2%. In the medical wards, the ICUs, and the surgery wards, *C. albicans* accounted for 66.4%, 54.8%, and 57.4% of the cases, respectively. The main difference came from *C. tropicalis* and *P. kudriavzevii*, particularly in hemato-oncology, where their proportions were higher than in other hospitalization units. On the other hand, *C. parapsilosis* prevalence was higher in internal medicine.

### 3.2. Species Distribution and Epidemiological Trends in Bloodstream Candida Infection

Yeasts were recovered from body fluids (51%), mainly peritoneal, biliary, and ascitic fluids. These were followed by blood cultures (28.3%), various tissues and biopsies (10%), and catheters (9.6%). (See Supplementary Table S3).

**Figure 3:**
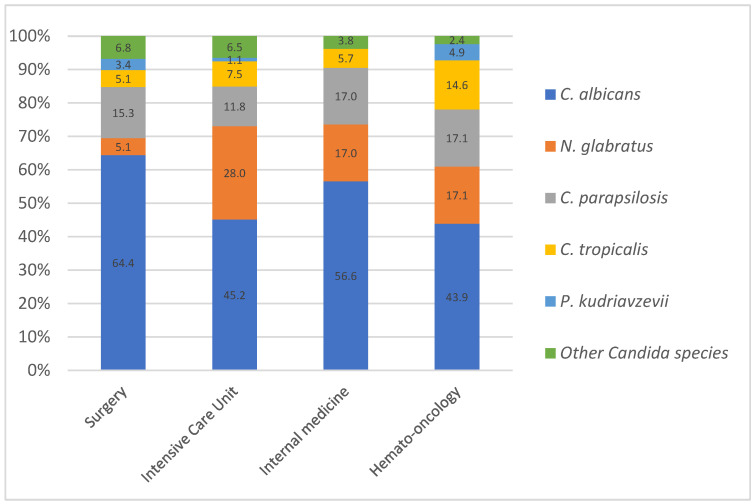
Distribution (%) of *Candida* species isolated from blood according to ward.

When focusing on candidemia, the distribution of *Candida* species differed from the overall distribution. The most frequently isolated species remained *C. albicans* (52%), followed by *N. glabratus* (18.5%), *C. parapsilosis* (14.7%), *C. tropicalis* (8.1%), *P. kudriavzevii* (1.9%), *C. guilliermondii* (1.5%), *C.dubliniensis* (1.2%), *C. kefyr* (0.7%), *C. lusitaniae* (0.7%), *C. inconspicua* (0.4%), and *C. pelliculosa* (0.4%). The proportion of *C. albicans* and NAC varied over time (see Supplementary Table S2). Although the ratio remained in favor of *C. albicans* over the 7 years, there was a shift in this ratio in 2019, 2021, and 2022, with the proportion of NAC at 54.4%, 51.5%, and 54.5%, respectively. The distribution of candidemia varied between wards (Figure 3). *C. albicans* showed the highest prevalence in surgery (64.4%) across all units combined and the lowest in hemato-oncology (43.9%) and ICUs (45.2%). *N. glabratus* had a notably high prevalence in ICUs (28.0%) compared to other units, and *C. tropicalis* remained the most prevalent in hemato-oncology (14.6%). 

### 3.3. Antifungal Susceptibility Patterns of Candida Species: Azole Resistance and Echinocandin Activity

Antifungal susceptibility testing was performed on all 916 isolates and is reported in Table 1. Susceptibility rates to fluconazole were 99.8% for *C. albicans* and 97.1% for *C. parapsilosis sensu stricto*. A decrease in fluconazole susceptibility was mainly observed for *N. glabratus*, which was intrinsically less susceptible, and for which 151 strains (89.9%) were dose-dependent susceptible (SDD). The overall prevalence of fluconazole resistance in the *N. glabratus* population was 10.1%, while the prevalence of non-wild-type (non-WT) isolates to voriconazole was 54.7%. Among these, two-thirds had an MIC at 0.5 mg/L, i.e., one dilution above the breakpoint. For *C. tropicalis*, susceptibility to azoles was decreased, with only 44 (67.7%) and 58 (89.2%) isolates showing susceptibility to fluconazole and voriconazole, respectively. Additionally, nine (81.8%) isolates exhibited cross-resistance to both fluconazole and voriconazole.

Finally, all *P. kudriavzevii* isolates showed high susceptibility to voriconazole (96%). Resistance to echinocandins was detected in 1 out of 25 strains (4%), while decreased susceptibility was observed in 13 out of 25 strains (52%). Among these, 1 strain (4%) exhibited decreased susceptibility to both anidulafungin and caspofungin, while 12 strains (48%) showed decreased susceptibility exclusively to caspofungin. The activity of the five antifungal agents tested against the uncommonly isolated species of *Candida* is detailed in Supplementary Table S4.

#### Fluconazole Susceptibility and Temporal Trends in *C. parapsilosis*

The fluconazole resistance rate for *C. parapsilosis sensu stricto*, across all samples, was 2.9%. The susceptibility profiles of these isolates, over the 7 years of the study period, are summarized in Table 2. Notably, no increase in resistance was observed over time; however, a decrease in the number of isolates was recorded in 2020, followed by a gradual increase over the past three years.

### 3.4. Isolated Detection of C. auris in a Single Imported Case

Only one set of samples containing *C. auris* was isolated in our center, in 2022, from a female patient who had sustained burn and trauma injuries, requiring an ICU stay in a Greek hospital before being transferred to our facility. This species was recovered from skin swabs but was not found in other invasive specimens and was therefore not included in this study.

## 4. Discussion

This study aimed to assess the epidemiology of *Candida* species in our hospital in light of recently reported resistance rates in *C. parapsilosis* [8,9]. Specifically, it sought to examine temporal shifts in species distribution, analyze differences across medical wards, and evaluate antifungal resistance patterns. The main objective was to assist clinicians in selecting optimal empiric antifungal therapy according to results. Treatment strategies can differ depending on the hospitalization unit and the underlying conditions. Current guidelines recommend echinocandins as a first-line treatment in most IC patients, mostly due to their broader spectrum of activity, higher fungicidal efficacy against the majority of *Candida* species, minimal drug interactions, low rates of acquired resistance, and favorable safety profile. Fluconazole is commonly used as a step-down therapy in cases where the *Candida* species is confirmed to be fluconazole-susceptible. However, guidelines for the use of fluconazole differ between major organizations. The Infectious Diseases Society of America (IDSA) guidelines recommend fluconazole as an alternative first-line treatment for non-neutropenic patients with no previous azole exposure. In contrast, the European Society of Clinical Microbiology and Infectious Diseases (ESCMID) guidelines, along with the recent global guidelines from the ECMM, do not recommend fluconazole as a first-line option for these patients, particularly in settings where antifungal resistance may be an issue [18]. Importantly, this recommendation from the IDSA does not extend to hemato-oncological patients, who frequently receive azole prophylaxis, which can lead to *Candida* infections with reduced sensitivity or natural resistance to fluconazole, such as *N. glabratus* and *P. kudriavzevii*, as well as acquired resistance. This may contribute to epidemiological trends and shifts in *Candida* species.

### 4.1. Temporal Distribution of Candida Species

Our findings reveal that *C. albicans* remained the predominant species, accounting for 56% of all isolates, followed by *N. glabratus*, *C. parapsilosis*, *C tropicalis*, and *P. kudriavzevii*. This distribution aligns with both European and global data, which show that despite a gradual rise in non-*albicans Candida* (NAC) species over the past decades, *C. albicans* continues to predominate [2,10,11].

When examining species distribution by sample type in our study, important differences emerged. In bloodstream infections (candidemia), *C. parapsilosis* ranked third after *C. albicans* and *N. glabratus*, a pattern also observed in a previous study in Belgium and Northern Europe [2,11,18,19]. Conversely, in other sterile-site samples—primarily peritoneal or ascitic fluids—*C. tropicalis* was more frequently isolated than *C. parapsilosis*. This discrepancy is biologically plausible: *C. parapsilosis*, which commonly colonizes the skin, exhibits strong adherence to medical devices and biofilm formation, particularly favoring catheter-related infections [2,5]. *C. tropicalis*, in contrast, is a commensal of the digestive tract and thus more likely to be found in intra-abdominal infections. Unfortunately, epidemiological studies investigating IC outside candidemia are lacking because of the absence of consensus on standardized definitions even after the publication of the FUNDICU project [4].

Several studies have observed a shift in the proportion of *C. albicans* toward non-*C. albicans Candida* (NAC) species over the past few decades. This shift may be linked to the aging population of immunosuppressed patients, the increased use of azole antifungals, and more frequent invasive procedures [12,19,20,21]. In our study, changes over time were highlighted. When considering all samples, the proportion of *C. albicans* remained stable (>50%) throughout the study period. However, when focusing specifically on candidemia, the ratio between *C. albicans* and NAC species fluctuated over time. A decreasing trend in *C. albicans* was observed, although it remained the predominant species. This finding aligns with other studies [2,11,12]. Both in candidemia and other invasive samples, there was a significant increase in *C. parapsilosis* and a decrease in *N. glabratus* proportions. These findings contrast with studies from Northern Europe, the US, and Australia, which have reported an increasing prevalence of *N. glabratus* [2,7,11,22,23,24,25]. *C. parapsilosis* has shown an increased proportion in Southern Europe, China, and Latin America [7,11,24]. These geographical variations may reflect differences in patient demographics, underlying risk factors, and antifungal treatment practices [26].

*N. glabratus* infections are primarily seen in elderly patients and solid organ transplant recipients, while *C. parapsilosis* is commonly associated with catheter colonization, neurosurgical procedures, polytrauma, and neonatal infections [2,5]. Although our study was not specifically designed to identify risk factors, the median age of affected patients remained stable at approximately 70 years over time, which does not fully explain the observed variation in species prevalence. Additionally, the apparent increase in *C. parapsilosis* detection may, in part, be influenced by sampling biases related to catheter-associated colonization rather than a true rise in invasive candidiasis incidence. Regarding antifungal exposure, fluconazole prophylaxis has been associated with a higher incidence of NAC infections, particularly *N. glabratus*, while the increased use of echinocandins has been linked to a reduction in *C. parapsilosis* candidemia but a concurrent rise in *C. tropicalis* infections [27]. Future studies in our center should focus on antifungal consumption stratified by ward and patient phenotype to look for these associations. Finally, the proportions of *C. tropicalis* and *P. kudriavzevii* have remained stable over time.

### 4.2. Distribution Across Medical Wards: Influence of Azole in High-Risk Patients

Our findings confirm that *C. albicans* remained the predominant species across all units; however, notable differences in species distribution were observed. In hematology-oncology wards, *C. albicans* was less prevalent compared to NAC species, with a significantly higher proportion of *C. tropicalis*. This observation aligns with previous studies highlighting the association between *C. tropicalis*, hematologic malignancies, and neutropenia [28,29]. These differences are likely related to local antifungal practices and patient-specific risk factors.

In our center, antifungal prophylaxis is administered to neutropenic patients: fluconazole is used in patients receiving autologous or allogeneic haematopoietic stem cell transplantation (HSCT) in the first month, while posaconazole is used during induction chemotherapy for acute myeloid leukemia and for GVHD. This may explain the higher prevalence of NAC species such as *C. tropicalis* in this population.

Moreover, in abdominal surgery patients, no antifungal prophylaxis is administered in our institution, in line with IDSA guidelines [30]. This could account for the stable predominance of *C. albicans* in surgical wards.

Focusing specifically on candidemia cases, we found that, in addition to hematology-oncology units, ICU patients also exhibited a lower proportion of *C. albicans* (43.9%) and a significantly higher prevalence of *N. glabratus* (28%). This aligns with findings by Lamoth et al., who reported that Candida infections in ICUs are often associated with prior fluconazole exposure, potentially contributing to shifts in species distribution [22].

### 4.3. Trends in Antifungal Resistance: A Focus on Candida Species and Emerging Resistance Patterns

Concerning susceptibility results, fluconazole and echinocandin resistance among *Candida* isolates remains low, except for a limited number of species such as *N. glabratus* and *P. kudriavzevii*. When it occurs, it is often associated with the long-term use of antifungals for treatment or prophylaxis [19,31].

In our study, the prevalence of resistance to azoles and echinocandins in *C. albicans* was less than 1%, and no resistance to amphotericin B was observed. This low rate of resistance is consistent with previous studies, particularly for fluconazole, where resistance rates between 0 and 3% are generally reported [11,31,32].

Similarly, our results indicate that *C. parapsilosis* has low resistance rates towards fluconazole, with 2.9% of resistant isolates. This is in line with previous reports estimating fluconazole resistance rates between 4% and 7% in *C. parapsilosis* [8,11,12]. However, in recent years, fluconazole-resistant strains of *C. parapsilosis* seemingly emerged during the COVID-19 pandemic. These clonal strains persist in various hospital niches, continually causing sporadic outbreaks in azole-naive patients, raising resistance rates to around 10% or even higher, especially in Southern Europe [3,8,9]. Recent studies highlight that these resistant strains are often associated with mutations in the *ERG11* gene, particularly the Y132F substitution, which confers high-level fluconazole resistance [13]. Moreover, novel resistance mechanisms, such as mutations in the *MRR1* gene and the overexpression of efflux pumps, have recently been described, further complicating treatment options and making these strains more difficult to manage [14].

Fortunately, we did not observe any increase in resistance in our hospital. Only two fluconazole-resistant strains were isolated in 2018 and 2022, but they could not be genotyped to determine the resistance gene(s) involved. Despite this concerning trend in certain regions of the world, resistance to echinocandins remains infrequent (0–3%), even though *C. parapsilosis* has a naturally higher minimum inhibitory concentration (MIC) due to an intrinsic polymorphism in FKS1 [12,31,32]. This suggests that echinocandins continue to be a viable treatment option for most infections caused by this species.

In contrast to the latter finding, the increasing antifungal resistance mostly observed in *N. glabratus* and *C. tropicalis* presents a growing clinical challenge.

For *N. glabratus*, even though this species is known for its intrinsically reduced sensitivity to fluconazole, we showed a high resistance rate of 10.1%. This rate is in line with previous studies reporting a range from 5.6% to 15.7% [31,32]. Furthermore, a recent European multicenter study showed that the highest rates were observed in the Czech Republic, Italy, Sweden, Turkey, the UK, and Belgium [2]. Cross-resistance between fluconazole and voriconazole is commonly observed in *Candida* species, and a similar pattern has been noted in *N. glabratus*, though not as direct resistance to voriconazole but through the presence of non-WT strains [11,31,32]. In *N. glabratus*, azole resistance is primarily attributed to the overexpression of drug efflux pumps CDR1, PDH1 (CDR2 in *C. albicans*), and SNQ2, with mutations in the *ERG11* gene occurring less frequently, as seen in other *Candida* species [27,33]. In our study, all fluconazole-resistant isolates were non-WT voriconazole, i.e., 10.1% of all *N. glabratus*. However, for the strains considered as sensitive to high doses of fluconazole, there was a significant proportion (44.6%) of non-WT voriconazole strains. This phenotype has already been documented in various studies, but there is currently no explanation for these findings [28]. Furthermore, despite being classified as susceptible to high doses of fluconazole, these strains still present elevated MIC values, reaching 16 mg/L and 32 mg/L. Therefore, voriconazole does not appear suitable in such cases, although current evidence remains insufficient to establish a clear correlation between in vitro susceptibility testing and clinical outcomes [34]. Concerning echinocandin resistance in *N. glabratus* in our study, the rate remained low (~1%), which is consistent with previous reports. Of note, some studies have noted an increase in resistance due to the selective pressure exerted by the widespread use of echinocandins [31,32,34].

Similarly, to *N. glabratus*, *C. tropicalis* demonstrated concerning resistance trends, with 16.9% of isolates resistant to fluconazole and 15.4% exhibiting reduced susceptibility. These rates exceed previously reported fluconazole resistance levels of 4–9% [22] but are consistent with findings from a prior Belgian multicenter study, which reported a fluconazole resistance rate of 20% [28]. High rates of resistance can also be observed in different countries, such as Taiwan and Australia, where rates reach 11% and 17%, respectively, which are also consistent with our results [25,26,35].

## 5. Conclusions

In our center, *C. albicans* remained the predominant species throughout the study period, with no significant shift favoring non-*albicans Candida* (NAC) species. An exception was observed in hemato-oncology, where antifungal prophylaxis contributed to the selection of a different distribution of species, notably with an increase in *C. tropicalis*.

Echinocandin resistance rates remained very low, confirming their efficacy as a first-line treatment. In contrast, azole resistance varied by species, with a marked increase observed in *N. glabratus* and *C. tropicalis*, which were particularly prevalent in high-risk units such as hematology-oncology. These findings highlight the need for careful antifungal management in these populations, favoring the empirical use of echinocandins in line with recent international guidelines, including those by ESCMID, Cornely et al. (2025) [18], and the IDSA.

Moreover, the low azole resistance observed in *C. albicans* and *C. parapsilosis*, along with the absence of a shift in prevalence towards non-*albicans* species, supports the empirical use of fluconazole in stable patients not previously exposed to azoles, consistently with IDSA guidelines.

Therefore, this study underscores the importance of species-specific antifungal susceptibility testing and ongoing local surveillance of resistance patterns to optimally tailor antifungal treatment strategies specific to at-risk patient populations.

## Figures and Tables

**Table 1:** Susceptibility of *Candida* isolates to antifungal drugs according to CLSI M27M44S clinical breakpoints or M57S epidemiological cutoff values.

	MIC Range (µg/mL)	MIC50 (µg/mL)	MIC90 (µg/mL)	Number of Isolates (%)			
				S/WT *	SDD	I	R/Non-WT **
*** C. albicans * (*n* = 509) **							
Amphotericin B	≤0.12–4	0.5	1	508 (99.8)	-	-	1 (0.2)
Fluconazole	≤0.12–16	0.25	1	503 (98.8)	1 (0.2)	-	5 (1)
Voriconazole	≤0.008–0.5	0.015	0.03	507 (99.6)		2 (0.4)	0
Itraconazole	-	-	-	-	-	-	-
Caspofungin	≤0.008–0.25	0.14	0.12	509 (100)	-	0	0
Anidulafungin	≤0.015–0.25	0.06	0.12	509 (100)	-	0	0
*** N. glabratus * (*n* = 168) **							
Amphotericin B	≤0.12–2	0.5	1	168 (100)	-	-	-
Fluconazole	0.25–≥256	16	32	-	151 (89.9)	-	17 (10.1)
Voriconazole	≤0.015–8	0.25	1	-	79 (45.3)	-	92 (54.7)
Itraconazole	0.5–16	0.5	1	-	160 (95.3)	-	8 (4.7)
Caspofungin	≤0.0008–8	0.12	0.12	161 (95.8)	-	5 (3)	2 (1.2)
Anidulafungin	≤0.015–2	0.12	0.12	167 (99.4)	-	0	1 (0.6)
*** C. parapsilosis sensu stricto * (*n* = 69) **							
Amphotericin B	≤0.12–2	0.25	0.5	69 (100)	-	-	-
Fluconazole	0.25–8	0.5	1	67 (97.1)	-	-	2 (2.9)
Voriconazole	≤0.015–0.25	0.03	0.12	68 (98.5)	-	1 (1.5)	-
Itraconazole	≤0.008–0.25	0.015	0.03	69 (100)	-	-	-
Caspofungin	0.12–4	0.5	1	67 (97.1)	-	2 (2.9)	0
Anidulafungin	0.12–4	1	2	65 (95.6)	-	4 (4.4)	0
*** C. tropicalis * (*n* = 65) **							
Amphotericin B	≤0.12–2	0.5	1	65 (100)	-		-
Fluconazole	0.25–≥256	0.25	4	44 (67.7)	10 (15.4)	-	11 (16.9)
Voriconazole	0.015–8	0.25	0.5	58 (89.2)	-	0	7 (10.8))
Itraconazole	0.03–8	0.12	0.5	57 (87.7)	-	1 (1.5)	7 (10.8)
Caspofungin	0.015–0.5	0.06	0.5	59 (90.7)	-	6 (9.3)	-
Anidulafungin	0.015–0.5	0.12	0.25	63 (96.9)	-	2 (3.1)	-
*** P. kudriavzevii * (*n* = 25) **							
Amphotericin B	≤0.25–2	1	2	25 (100)	-		-
Fluconazole	8–128	64	128	-	-	-	25 (100)
Voriconazole	0.12–1	0.5	0.5	24 (96)	-	1 (4)	
Itraconazole	0.12–1	0.5	0.5	24 (96)	-	-	1 (4)
Caspofungin	0.12–1	0.5	0.5	11 (44)	-	13 (52)	1 (4)
Anidulafungin	≤0.015–4	0.06	0.12	24 (96)	-	1 (4)	-

Abbreviations: CLSI, Clinical and Laboratory Standards Institute; I, intermediate; R, resistant; S, susceptible; SDD, susceptible, dose-dependent; WT, wild type. In the absence of established CLSI clinical breakpoints, CLSI epidemiological cutoff values (ECVs) were used for discrimination between WT * and non-WT ** isolates (CLSI document M57S). MIC50 (minimum inhibitory concentration for 50% of isolates), MIC90 (minimum inhibitory concentration for 90% of isolates).

**Table 2:** Trends in fluconazole resistance in *Candida parapsilosis* over 7 years.

Antifungal	Susceptibility	2017	2018	2019	2020	2021	2022	2023
Fluconazole	S	7/7 (100%)	4/4 (100%)	11/12 (91.7%)	6/6 (100%)	11/11 (100%)	13/14 (92.9%)	15/15 (100%)
	SDD	0	0	0	0	0	0	0
	R	0	0	1/12 (8.3%)	0	0	1/14 (7.1%)	0

## Data Availability

The original contributions presented in this study are included in the article/Supplementary Material. Further inquiries can be directed to the corresponding author.

## Supplementary Materials

The following supporting information can be downloaded at https://www.mdpi.com/article/10.3390/jof11060465/s1. Table S1: Distribution of *Candida* species over 7 years of surveillance; Table S2: Distribution of *Candida* species isolated from candidemia; Table S3: *Candida* species recovered from clinical samples; Table S4: Activity of 5 antifungal agents tested against 9 uncommonly isolated species of *Candida*.
