# Supplementary material for: Species Distribution and Antifungal Susceptibility Patterns of Invasive Candidiasis in a Belgian Tertiary Center: A 7-Year Retrospective Analysis
Source: J Fungi (Basel). 2025 Jun 19;11(6):465. doi: 10.3390/jof11060465 (PMC12194159; doi:10.3390/jof11060465)
Supplement: Supplementary file 1 [file jof-11-00465-s001.zip › jof-3577354-supplementary.pdf]

Supplementary Materials

Table S1. Distribution of *Candida* species over 7 years of surveillance

	2017	2018	2019	2020	2021	2022	2023	Overall
Species	No. (%)	No. (%)	No. (%)	No. (%)	No. (%)	No. (%)	No. (%)	No. (%)
C. albicans	74 (55,6)	91 (58,7)	79 (58,1)	70 (57,4)	72 (55,8)	76 (53,9)	56 (56)	518 (56%)
N. glabratus	37 (27,8)	36 (23,2)	26 (19,1)	23 (18,9)	12 (9,3)	25 (17,7)	15 (15)	174 (19%)
C. parapsilosis	7 (5,3)	4 (2,6)	12 (8,8)	6 (4,9)	11 (8,5)	15 (10,6)	15 (15)	70 (8%)
C. tropicalis	10 (7,5)	11 (7,1)	5 (3,7)	9 (7,4)	12 (9,3)	11 (7,8)	7 (7)	65 (7%)
P. kudriavzevii	2 (1,5)	5 (3,2)	3 (2,2)	4 (3,3)	7 (5,4)	4 (2,8)	0	25 (3%)
C. dubliniensis	1 (0,8)	3 (1,9)	5 (3,7)	4 (3,3)	3 (2,3)	3 (2,1)	3 (3)	22 (2%)
C. kefyr	2 (1,5)	2 (1,3)	3 (2,2)	5 (4,1)	2 (1,6)	2 (1,4)	2 (2)	18 (2.6%)
C. lusitaniae				1 (0,8	4 (3,1)	2 (1,4)	2 (2)	9 (1%)
C. guillermondii		1 (0,6)	1 (0,7)		3 (2,3)	2 (1,4)		7 (0.7%)
C. inconspicua		2 (1,3)	1 (0,7)		1 (0,8)			4 (0.4%)
C. parrugosa			1 (0,7)					1 (0.1%)
C. metapsilosis						1 (0,7)		1 (0.1%)
C. norvegiensis					1 (0,8)			1 (0.1%)
C. pelliculosa					1(0,8)			1 (0.1%)

Table S2. Distribution of *Candida* species isolated from candidemia

	2017	2018	2019	2020	2021	2022	2023	Total (n, %)
C. albicans	16	24	15	16	16	20	27	134 (52%)
N. glabratus	9	5	8	5	3	8	10	48 (16,5%)
C. parapsilosis	4	2	7	2	7	6	11	39 (14,7%)
C. tropicalis	2	5	1	4	3	4	2	21 (8,1%)
P. kudriavzevii	0	2	0	1	0	2	0	5 (1,9%)
C. dubliniensis	0	0	1	0	1	0	1	3 (1,2%)
C. kefyr	1	0	0	0	0	1	0	2 (0,7%)
C. lusitaniae	0	0	0	0	0	1	1	2 (0,7%)
C. guillermondii	0	0	1	0	1	2	0	4 (1,5%)
C. inconspicua	0	0	0	0	1	0	0	1 (0,4%)
C. pelliculosa	0	0	0	0	1	0	0	1 (0,4%)
	32	38	33	28	33	44	52	260

Table S3. *Candida* species recovered from clinical samples.

Species	Blood	Body fluids								Catheter	Material		Various tissues and biopsies			
		Peritoneal fluid	Abscess	Ascitic fluid	Bile	Drain	Pus	Pleural fluid	Synovial fluid		Biliary prosthesis	Other material	Biopsy	Bone	Lithiasis	Others
C. albicans	134	97	17	30	60	23	39	13	2	67	4	5	14	8	2	13
N. glabratus	48	32	9	16	17	13	12	4		9		1	7	2	2	2
C. parapsilosis	39			1	2		2		2	8	1	1	4	9	1	1
C. tropicalis	21	8	2	9	6	4	4	1	1		1	1	4			3
P. kudriavzevii	5	5		3		3	2	4					1			2
C. dubliniensis	3	7	2	1	1	3	1			3	2					
C. kefyr	2	1		3	4		1	1		1	1	1			2	
C. lusitaniae	2	1			1	1	3						1			
C. guillermondii	4	1		1									1			
C. inconspicua	1			2								1				
C. pelliculosa	1															
C. metapsilosis						1										
C. norvegensis		1														
C. parrugosa														1		
	260	153	30	66	91	48	64	23	5	88	9	10	32	20	7	21

Table S4. Activity of 5 antifungal agents tested against 9 uncommonly isolated species of *Candida*

			MIC, mg/L		
**Species**	No.Tested	Antifungal Agent	Range	MIC50	MIC90
C. dubliniensis	22	Amphotericin B	≤ 0.12-0.75	0.25	0.5
		Floconazole	≤ 0.12-16	0.25	1
		Itraconazole	≤ 0.015-1	0.03	0.12
		Caspofungin	0.03-0.12	0.12	0.12
		Anidulafungin	0.03-0.25	0.12	0.12
C. kefyr	18	Amphotericin B	0.25 - 2	1	1
		Fluconazole	≤ 0.12-16	0.5	2
		Itraconazole	0.03 – 0.5	0.12	0.5
		Caspofungin	0.015 – 0.5	0.03	0.12
		Anidulafungin	0.12 – 0.25	0.12	0.25
C.lusitaniae	9	Amphotericin B	0.25-0.5	0.5	0.5
		Fluconazole	0.5 - 64	1	2
		Itraconazole	0.06 – 0.5	0.12	0.25
		Caspofungin	0.03 – 0.25	0.25	0.25
		Anidulafungin	0.015 – 0.5	0.12	0.5
C.guillermondii	7	Amphotericin B	≤ 0.12-1	0.25	1
		Fluconazole	1 - 32	4	8
		Itraconazole	0.12 - >16	0.5	0.5
		Caspofungin	0.12 - 0.5	0.25	0.5
		Anidulafungin	0.5 – 2	1	4
C.inconspicua	4	Amphotericin B	0.12 – 1.0	0.5	-
		Fluconazole	0.5 – 32	4	-
		Itraconazole	0.03 – 0.5	0.12	-
		Caspofungin	0.06 – 0.25	0.12	-
		Anidulafungin	0.015 – 0.12	0.06	-
C.pararugosa	1	Amphotericin B	0.25	-	-
		Fluconazole	2.0	-	-
		Itraconazole	0.03	-	-
		Caspofungin	0.12	-	-
		Anidulafungin	0.06	-	-
C.metapsilosis	1	Amphotericin B	0.5	-	-
		Fluconazole	1.0	-	-
		Itraconazole	0.06	-	-
		Caspofungin	0.06	-	-
		Anidulafungin	0.12	-	-
C.novenginiensis	1	Amphotericin B	1.0	-	-
		Fluconazole	32	-	-
		Itraconazole	0.25	-	-
		Caspofungin	0.12	-	-
		Anidulafungin	0.06	-	-
C.pellicolosa	1	Amphotericin B	0.12	-	-
		Fluconazole	> 8.0	-	-
		Itraconazole	0.25	-	-
		Caspofungin	0.015	-	-
		Anidulafungin	0.015	-	-
